# Causality and correlation analysis for deciphering the microbial interactions in activated sludge

**DOI:** 10.3389/fmicb.2022.870766

**Published:** 2022-08-04

**Authors:** Weiwei Cai, Xiangyu Han, Thangavel Sangeetha, Hong Yao

**Affiliations:** ^1^School of Civil Engineering, Beijing Jiaotong University, Beijing, China; ^2^Research Center of Energy Conservation for New Generation of Residential, Commercial, and Industrial Sectors, National Taipei University of Technology, Taipei, Taiwan; ^3^Department of Energy and Refrigerating Air-Conditioning Engineering, National Taipei University of Technology, Taipei, Taiwan

**Keywords:** network, causality, microbial community, activated sludge, correlation

## Abstract

Time series data has been considered to be a massive information provider for comprehending more about microbial dynamics and interaction, leading to a causality inference in a complex microbial community. Granger causality and correlation analysis have been investigated and applied for the construction of a microbial causal correlation network (MCCN) and efficient prediction of the ecological interaction within activated sludge, which thereby exhibited ecological interactions at the OTU-level. Application of MCCN to a time series of activated sludge data revealed that the hub species OTU56, classified as the one belonging to the genus *Nitrospira*, was responsible for nitrification in activated sludge and interaction with *Proteobacteria* and *Bacteroidetes* in the form of amensal and commensal relationships, respectively. The phylogenetic tree suggested a mutualistic relationship between Nitrospira and denitrifiers. *Zoogloea* displayed the highest *ncf* value within the classified OTUs of the MCCN, indicating that it could be a foundation for activated sludge through the formation of characteristic cell aggregate matrices where other organisms embed during floc formation. Inclusively, the research outcomes of this study have provided a deep insight into the ecological interactions within the communities of activated sludge.

## Introduction

Ecological interactions, such as those involved in the exchange of resources or space, within microbial communities have been topics of intense interest in microbial ecology (Hibbing et al., 2010). The interactions of species are considered a driving force promoting ecological functions of the microbial communities, and due to its importance, the structure of communities has been described by species interaction networks for over a century (Berlow et al., 2009; Poisot et al., 2015). Although networks were initially applied to the study of food webs, the concept has been expanded to microbial ecology to unravel ecological interactions (Ings et al., 2009; Kéfi et al., 2012). Therefore, microbial interactions within a community are more likely to be reflected by network theory, which can be established through a set of methodologies by mathematical correlations. Recently, network theory has been commonly used to explore the microbiomes of natural and artificial environments, such as soil (Barberan et al., 2012), sediments (Ji et al., 2016), bioreactors (Liang et al., 2018), and wastewater treatment plants (Global Water Microbiome Consortium et al., 2019).

In wastewater treatment plants, activated sludge has served as the core unit for wastewater treatment for decades (Jenkins and Wanner, 2014). The highly diverse microorganisms in activated sludge thrive on organic compounds that are enriched in carbon (C), nitrogen (N), sulfur (S), phosphorus (P), and various trace elements, forming a complex web of ecological interactions based on the competition for resources and space (Liébana et al., 2016; Xia et al., 2018). A series of graphical methods have been developed for the construction of correlation or co-occurrence networks, visualization, and elucidation of the complex microbial interactions of species in the activated sludge, gut microbiome, and natural environment (Weiss et al., 2016). Previous studies on co-occurrence or correlation networks have defined multiple relationships between species with a pairwise similarity matrix or sparse multiple regression analysis, respectively (Faust and Raes, 2012). Generally, nodes and links in a network, respectively, represented species and interactions, yet, these interactions were only defined by positive or negative association, which limited further understanding of ecological interactions between species. As an intrinsic property of correlation analysis, previous networks were commonly undirected, demonstrating specific interactions among species, such as competition and symbiosis. Although a few studies have attempted directed networks, provided according to the time lag, to show a direction between nodes (Ju and Zhang, 2015; Deng et al., 2016), most studies have rarely explored the possibility of causality analysis from time-series data, which could enhance our understanding of ecological interactions.

Consequently, Spearman’s correlation and Granger test were implemented to infer the correlation and causality between members of an activated sludge microbiome. The causality pointed out the direction of interaction, whereas, the correlation displayed whether it was positive or negative. Later, the combination of correlation and causality was used to reflect the ecological interaction. A previously published 259-day high-through sequencing data set was employed for correlation analysis and the Granger test (Jiang et al., 2018). Coupling the correlation and causality analyses allowed the construction of a microbial causal correlation network (MCCN), which demonstrated that the microbial interactions in activated sludge could be classified as mutualism, synergism, commensalism, neutralism, predation (parasitism), amensalism, and competition (antagonism). Hub-species OTU56 belonged to *Nitrospira* and showed more diverse interactions with *Proteobacteria* as compared to *Bacteroidetes*. Moreover, the *Zoogloea* was potentially the key genus that induced changes in many of the activated sludge bacteria due to their role in scaffold construction during sludge floc formation. The application of MCCN will provide information on the ecological interactions between different species in both natural and artificial ecosystems.

## Results and discussion

### Applicability of granger causality

The assembly of the microbial community is commonly recognized as the result of deterministic and stochastic processes. The role of deterministic processes has been observed to be limited in stable environments, where their stochasticity could play an important role in gradually shifting community structure (Zhou and Ning, 2017). Due to the mutual influence of both processes, the relative abundance of a specific species is assumed to be the sum of a baseline and random variation. As the variation of species includes the random section, the random variation of species can be a joint distribution over time. The present microbial community has its evolution from the previous state, while time should have limited influence on the variation of the microbial community due to the presence of stochasticity. Although past observations are significant to forecast future trends, these predictions are not entirely dependent on them. Therefore, there could be an autocorrelation process, which might produce a time lag representing only finite past values for forecasting. Deng et al. (2016) used the time lag to construct the correlation network with time-series data and unravel microbial succession within a uranium bioremediation site (Deng et al., 2016). Additionally, David et al. (2014), when analyzing the effect of host lifestyle on human microbiota, relied on the autocorrelated process of time series (David et al., 2014), which demonstrated that OTUs’ variation complied with the time series model. We applied the data of 98 key OTUs obtained over the course of 259 days to fit into the augmented Dickey–Fuller (ADF) test to verify whether microbial data was irrelevant to time or not. If the data was not stationary, it indicated that the time series data were independent of time and the difference between the adjacent values will be applied to all data. The results of the stationary check are shown in Supplementary Data S2. All OTUs fulfilled the requirement of stationary after difference, 51 OTUs required difference treatment while the rest were stationary without it.

### Overall topological indexes of the causal network

**FIGURE 1:**
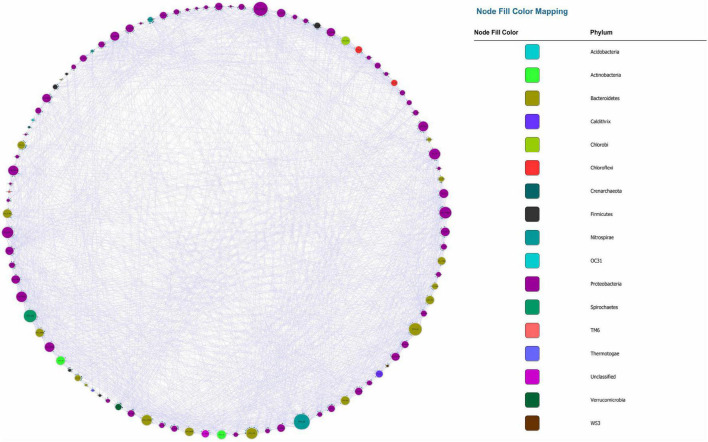
Microbial Granger Causal Network, each color represents a separate phylum. The size of the node and node label is proportionate to the edge number of each node from 0 to 110. The arrows represent the direction of Granger causality.

The visualized causal network is shown in Figure 1. A total of 98 OTUs were used for Microbial Granger Causal Network (MGCN) construction, which created 1,865 links between the nodes at a significant threshold of *p* < 0.05. Granger causality is commonly non-symmetric, and network building and the bidirectional links were defined as feedback from the source to the target OTU, indicating that either node could improve the forecasting accuracy of the other. A unidirectional link indicated that the source OTU significantly improved the forecasting accuracy of the target OTU but not vice versa. The outdegree and indegree directed links, defined by the direction of links in or out of the specific node, were counted separately. As revealed in Table 1, the distribution of the nodes degree tended to be normal rather than following power-law, regardless of whether indegree or outdegree, implying that the causal network was not scale-free (Deng et al., 2012).

**TABLE 1:** Properties of different networks.

	Granger		Random		Spearman		Random		Random	
	MGCN	BoMGCN	MGCN	BoMGCN	MCN	BoMCN	MCN	BoMCN	MCCN	MCCN
Network size	98	81	98	81	98	98	98	97	73	73
Network density					0.811	0.568	0.811	0.568		
Links	1856	730	1856	730	3856	2644	3856	2644	441	441
Power law (in)	0.041	0.468	0	0.284	0.211	0.101 (in total)	0.292	0.02	0.552	0.016
Power law (out)	0.03	0.064	0.043	0					0.595	0.111
Average clustering coefficient	0.449	0.373	0.196	0.119	0.868	0.753	0.812	0.57	0.352	0.084
Network diameter	6	8	3	4	2	4	2	2	7	5
Network radius	3	1	2	3	2	2	2	2	1	3
Average shortest paths	2.149	2.647	1.823	2.21	1.189	1.463	1.189	1.432	2.866	2.571
Average number of neighbors	27.082	14.716	34.143	17.21	78.694	54.515	78.694	54.515	9.753	11.507
*cd*	0.098	0.056	NA		NA		NA		0.042	NA
ge	0.815	0.9							0.93	
Average *cs*	0.491	0.504							0.509	
Average *c*_*recip*_	0.238	0.148							0.157	

NA represents there is no data for this property.

The average clustering coefficient, which reflected the clustering degree of the overall network, was defined as the average of the clustering coefficients of all the nodes. The clustering coefficient of MGCN (0.449) was higher than previously described undirected networks, including grassland soils (0.1–0.22), lake sediment (0.09), and groundwater condition (0.17–0.29), and was comparable with the value of 0.466 observed in a previous activated sludge study (Ju and Zhang, 2015). Watts and Strogatz (1998) introduced the random rewiring procedure to interpolate regular and random networks, in which the regular lattice was highly clustered while the random network was poorly clustered (Watts and Strogatz, 1998). Therefore, the higher relative clustering exhibited by causality indicated that the network was defined rather than random. The average shortest average path was 2.149, which was smaller than that within the undirected network. Hence, we derived a relatively clustered network connected by shorter paths, demonstrating that the neighboring nodes were closely connected. To confirm the small-world property, randomized networks with the same nodes and degrees as the original network were constructed. The average clustering coefficient and shortest paths were ∼0.196 and 1.823, respectively, whereas the ratio of the Granger network to the random network of clustering coefficient and shortest path can be determined (Liao et al., 2011). The ratio was equal to ∼1.943, and this indicated that the network possessed small-world properties.

### Indexes of nodes

**FIGURE 2:**
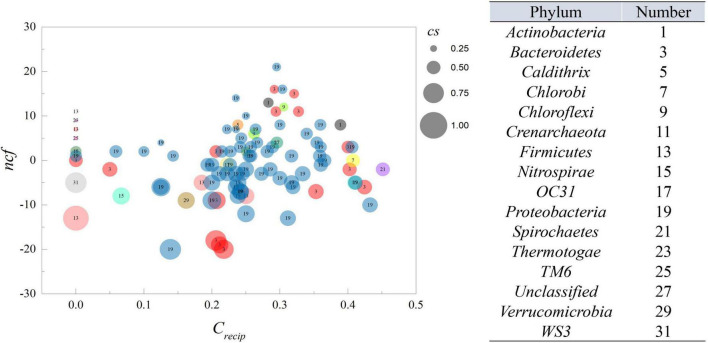
The *ncf* and *c*_*recip*_ from MGCN. Each circle represents an individual node from MGCN with size representing the *cs* value. The number within the circle corresponds to the classification of OTU at the phylum level.

According to the definition of *cs*, its magnitude represents the ability of a specific OTU to cause variation among its neighbors. A value of 1 indicates that an OTU can affect its neighbors without being affected by them, while zero indicates the opposite. The *c*_*recip*_ reflected counts of reciprocating links, which exhibited feedback behavior of each OTU, thereby higher values indicated that an OTU is likely to interact with others. Therefore, as shown in Figure 2, as *c*_*recip*_ increased, the *cs* inclined to approach 0.5, displaying an equilibrium of indegree and outdegree links. All nodes displayed a *c*_*recip*_ value of less than 0.5, suggesting bidirectional links were not dominant in the relationship of all nodes. However, it was interesting that more interactions could be positively related to the trend of *cs*. The *cs* and *c*_*recip*_ were both relatively quantified as the proportion excluded the magnitude of degrees, and node size in Figure 2 is proportional to the degree of connection with the neighboring nodes. The average number of neighbors for a node was ∼27.08. The majority of nodes with a large number of neighbors had a higher *c*_*recip*_ and neutral position of *cs*. Nodes with lower *c*_*recip*_ and lower *cs* indicated that more links were indegree, with the reverse, higher *c*_*recip*_ and *cs* indicating more links were outdegree. Integration of the relative proportion and neighbor number, which was considered as an absolute quantity, was beneficial for inferring the central output nodes in the network, which should possess lower *c*_*recip*_, higher *cs*, within a fairly large size of neighbors. The average of *cs* was ∼0.491, showing that the number of outdegree and indegree links were nearly identical. The average of *c*_*recip*_ was 0.24, implying mutual cause is not predominant due to the lower proportion in total links. Additionally, *ncf*, the difference between net outdegree and net indegree, of nodes ranged from −20 to 21, as shown in Supplementary Data S3. The average individual outdegree was 8.14 and the average net indegree was the same. Moreover, the number of OTUs with positive *ncf* was greater than that of negative *ncf*, indicating more than 50% of the relationships in the system displayed Granger causality in the activated sludge system.

### Bonferroni-correction

**FIGURE 3:**
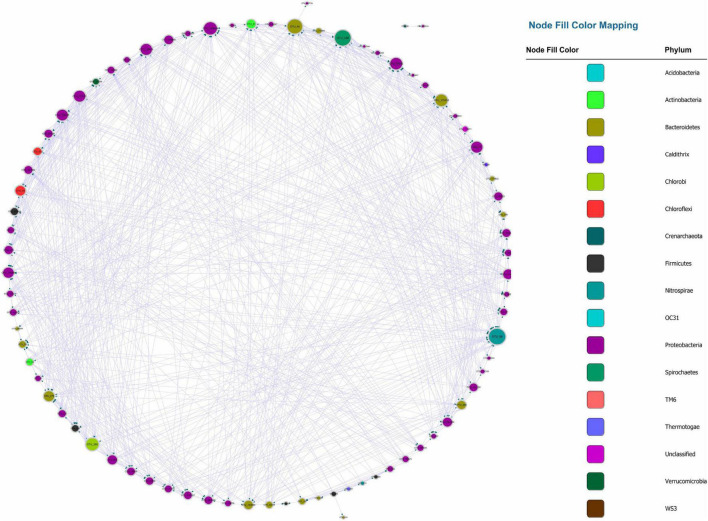
Bonferroni-corrected MGCN, each color represents a separate phylum. The size of the node and node labels are proportionate to the edge number of each node from 0 to 110. The arrows represent the direction of Granger causality.

Recently, numerous studies have used Bonferroni-correction to improve the threshold of correlation prediction (Navarrete et al., 2015; Quinn et al., 2016; Castellanos et al., 2020). The Bonferroni-corrected MGCN (BoMGCN) was produced from the original significant network (Figure 3). The corrected network was sparser in comparison with the causal network, containing only 81 nodes and 730 links and a lower clustering coefficient (0.373). The reduced network was highly conservative as the Bonferroni-correction excludes all potential type I error (false link was accepted) and displayed a slightly improved stability as revealed by the R square of power-law. The value of outdegree R square was 0.064, close to zero, yet the value for indegrees was 0.468, indicating a significant increase. Although the values were too trivial to be fitted into power law, they indicated that some nodes in the BoMGCN had a greater or lesser effect on other nodes. An improvement of the scale-free property was also observed, as well as an increase in the small world index represented by an increase in the ratio of σ (∼2.617). This was caused by a decrease in the clustering coefficient and an increase in the average shortest path, indicating that the BOGCN was more likely to fall under the rules of a small world. Within the random network derived from BOGCN there was a clear decrease in the clustering coefficient (0.119). Additionally, the properties of total nodes were slightly distinguished from the original MGCN network as a clear decline of *cd* value was noticed. The average *cs* increased from 0.491 to 0.504. Overall, the BoMGCN reduced the size of the network while retaining its basic properties. According to the classification of OTUs in BoMGCN, *Proteobacteria* was the predominant node and the hub species was *Nitrospira*, indicating the nitrogen-associated species had a broader social connection with other microbes.

### Correlation-network supplemented to causal network

**FIGURE 4:**
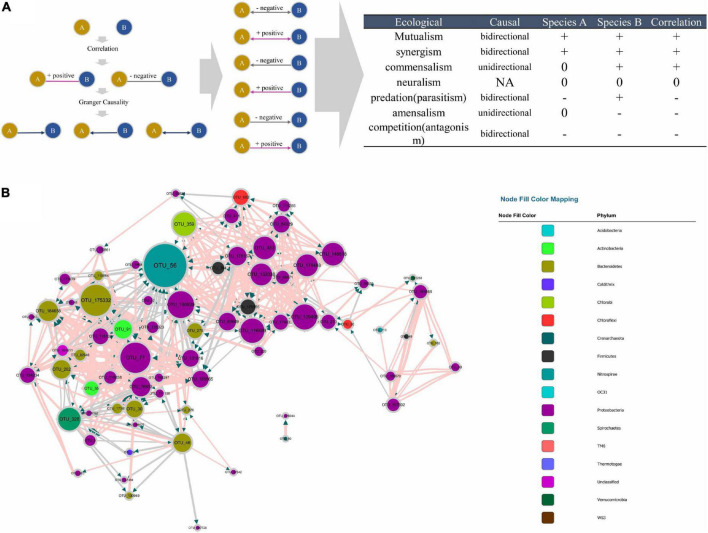
**(A)** Principle of the MCCN inference from the combination of causality and correlation, the detail of correspondence from MCCN link to ecological interaction on the right table. **(B)** MCCN, each color represents an individual phylum. The size of the node and node labels are linearly proportionate to the edge number of each node from 0 to 50. The arrows represent the direction of Granger causality. Pink and gray link colors represent positive and negative associations, respectively. The size of the link is proportionate to the correlation absolute value from 0 to 1.

The MGCN disclosed the casual effect within the microbial community; however, information about positive or negative correlations between nodes was missing. Therefore a Bonferroni-corrected microbial correlation network (BoMCN) based on Spearman’s correlation (shown in Supplementary Data S4) was applied to supplement the MGCN, constructing the MCCN. The multiple relationships between two OTUs could be revealed more explicitly according to this combination of causality and correlation. Previously, correlation analysis was generally used to discern the negative and positive relations within a microbial network, indicating the ecological interactions between members of the community (Faust and Raes, 2012). The Granger test was also recently applied in the research of a microbial network (Ai et al., 2019; Mainali et al., 2019), but the positive or negative effects could not be predicted with a single method. As shown in Figures 4A, a combination of correlation and Granger causality could be used to construct a new relationship, which indicated the directional connection among nodes, including the positive or negative effect they had on each other. As displayed in Figure 4B, the MCCN was composed of 73 nodes and 441 links. Although the causality was at a higher level compared with correlation, i.e., all nodes with causal links indicated a strong mutual interaction, the missing nodes and links could be ascribed to the Granger causality, which was not a real causal relationship, due to the limitations of the method. Technically, the Granger test has been widely used for predicting the causal effect. In the context of the current study, the Granger test was utilized to forecast the relations between OTUs, which may lead to a better understanding of microbial behaviors and relationships within a community. But additional efforts would be required to verify interactions between species.

The combination of correlation and Granger test allowed the observation of more specific interactions between the two species, therefore a MCCN network could be applied to predict ecological relationships for community analysis. As shown in Figure 4A, there are seven patterns of species interactions, including mutualism, synergism, commensalism, neutralism, predation (parasitism), amensalism, and competition (antagonism) (Pepper et al., 2015). According to the results of MCCN, both mutualism and synergism should be a bidirectional edge with a positive effect on both species, as each species would derive benefits from the other, such that it would be difficult to distinguish them apart. Commensalism can be reflected by a unidirectional link with a positive effect as species A can obtain a metabolite produced by species B. Although species B would be irrelevant to the growth of species A, as there is no feedback from A to B, the sequencing data of the two species would be positively correlated as more species B would secrete more metabolites for species A. Oppositely, a unidirectional connection with negative effect is classified as amensalism due to the general release of inhibitors from species A to species B. Here, the quantity of species A will be relevant to the production of inhibitors, such as antibiotics, which can reduce the number of species B, thereby the contrary growth of species A and B will lead to a negative correlation. Although the predation (parasitism) can be implied by the negative bidirectional edge, the sequencing data used in this study contained only information from the 16S rRNA gene of bacteria, with no information about protozoa or phages, resulting in the exclusion of predation (parasitism) from the MCCN of the microbial community (Deng et al., 2016). Finally, a negative bidirectional link could also indicate competition between species. The MCCN is a powerful tool to recognize multiple interactions of microbes by specifying the endogeneity of correlation, which has been widely used as a statistic proof of microbial interaction within a network (Weiss et al., 2016).

### Core species in microbial causal correlation network

**FIGURE 5:**
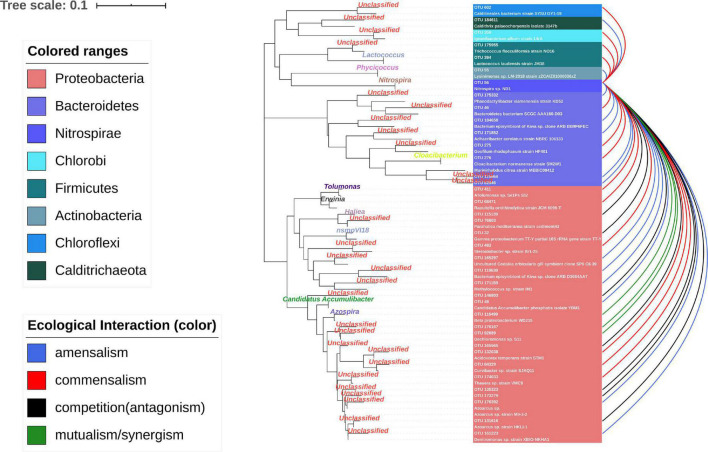
Ecological interaction of OTU56 with others at the OTU level. The color represents the type of interaction. The phylogenetic tree shows the closest species according to the results of the NCBI blast.

The nodes with amounts of links would be considered “hubs” in the MCCN. OTU56 was the hub species with the greatest number of indegrees (31) and the second highest number of outdegrees (16). It was classified as genus *Nitrospira*, a globally distributed group of nitrite oxidizers (NOB), which was also verified to be able to achieve complete nitrification from ammonia to nitrate in one step (van Kessel et al., 2015). As shown in Figure 5 and Supplementary Data S5, S6, OTU56 closely interacted with 24 OTUs from the phylum *Proteobacteria*, 8 OTUs from the phylum Bacteroidetes, and the remaining 6 OTUs interacted with 5 additional phyla. A total of 21 OTUs displayed negative interactions with *Nitrospira*, of which 14 were amensalism and 7 were competition relationships. Interestingly, all competition interactions originated from *Proteobacteria* to *Nitrospira*, showing that a number of *Proteobacteria* may depress the growth of *Nitrospira*. This could be ascribed to the fact that most bacteria related to the nitrogen cycle were *Proteobacteria* (Costa et al., 2006). Additionally, OTU56 unidirectionally interacted with OTUs from *Bacteroidetes*, for which there were only two types of interactions, commensalism and amensalism, with 3 and 5 links, respectively. According to a global diversity and biogeography study of over 300 wastewater treatment plants, only 28 out of 61,448 OTUs, accounting for 12.4% of the 16S rRNA gene sequences, were defined as core OTUs, and these mainly consisted of *Proteobacteria*, *Bacteroidetes*, and *Nitrospira* in activated sludge (Global Water Microbiome Consortium et al., 2019). Therefore, the results of MCCN in this study were consistent, as *Proteobacteria* and *Bacteroidetes* actively interacted with the core species of *Nitrospira*, a group that played the crucial role in nitrification of the activated sludge. At the genus level, the majority of species that interacted with OTU56 were unclassified, and of those that could be identified, *Azospira*, which possesses denitrification activity, exhibited a mutualistic relationship with *Nitrospira*, as well as with OTU176167 and OTU92689, which were most closely related to the genus *Dechloromonas*, members of which are capable of reducing nitrate or chloride. The above mutualistic relationships could be achieved in nitrogen cycling processes, with denitrification removing nitrate as a product inhibitor to *Nitrospira*, meanwhile, *Nitrospira* could supply nitrate as a substrate for denitrifies.

OTU180929, which had the most outdegree links (17), was classified as *Sinobacteraceae* at the family level. Members of this family are known to play a role in the degradation of aliphatic and aromatic hydrocarbon compounds and small organic acids (Gutierrez et al., 2013; Zhang et al., 2018). The number of net outdegrees and net indegree indicated the trending of nodes to cause a change of others or be affected by others. OTU180929, belonging to the genus *Zoogloea*, possessed 13 net outdegree and 13 net indegree links separately. *Zoogloea* has previously been demonstrated to be a bacterial genus important in the process of floc formation (Shao et al., 2009), and in this study it is represented by OTU180929 and OTU178488. As shown in Supplementary Data S7, the *ncf* of *Zoogloea* had the highest value within the sum of classified OTUs, indicating that *Zoogloea* could enhance the growth of most species, i.e., it could be the foundation for the formation of activated sludge. However, the *ncf* of unclassified OTUs was still higher, reaching 18. The culture-dependent methods build the basics of microbiology research, which investigate the role of specific species (mostly filamentous) in sludge flocculation and foaming (Nielsen et al., 2009). The unclassified nodes in MCCN notified that a massive microbial dark matter remained in activated sludge that was not yet cultured. The network approach has been used to elucidate and prioritize the microbial dark matter in the microbial community (Zamkovaya et al., 2021). Although activated sludge has been a widely employed strategy in wastewater treatment plants for over 100 years (Nielsen and McMahon, 2014), its microbiome still contained many mysterious and abundant unknown species that are only gradually being elucidated by recent progress in culture-dependent and independent technologies. Although our results showed the ecological interaction in the microbial community according to their variation with time, the Granger and correlation test only depend on the time-series data, which still needs experimental validation to verify the specific metabolic dependency between different species.

In conclusion, the coupling of correlation and causality was crucial to understand the ecological interactions within the microbial community. The MCCN disclosed a sophisticated causal network in activated sludge and identified the fundamental species, with the highest *ncf* value, as *Zoogloea*. The MCCN and phylogenetic analysis together indicated that the core-species of *Nitrospira* (OTU56) could have mutualistic interactions with denitrifiers in activated sludge. However, most species that interacted with OTU56 were still unclassified, implying a greater sequencing depth would be the key to improve the understanding of activated sludge.

## Materials and methods

### Sequencing data derivation

The sequencing data were acquired from NCBI (accession number: PRJNA324303), which has been published previously (Jiang et al., 2018). The time-series data set included sequencing data for 259 days taken from a long-term operational wastewater treatment plant. The primers were F515 and R806, which covered mostly bacteria and archaea. The achieved fastq files were combined and processed online using a galaxy platform (Feng et al., 2017). OTUs were created with 97% cut-off through the Uparse clustering method. RDP classifier assigned one representative sequence from each OTU to bacteria or archaeal taxonomy according to the 16S rRNA Greengene Database. The final OTUs table was prepared for the subsequent process. The phylogenetic tree was created with Mega software with N-J method and the visualization was completed online^1^ (Letunic and Bork, 2019).

### Stationarity

Stationarity is an important concept for time series analysis and is a precondition to Granger causality. The properties of stationarity were defined by three main factors in terms of mean, variance, and covariance. The stationarity indicated that there was no change of trend in the time data, and it was known as a changeless process of the joint distribution within a specific displacement. The stationary implied that the expectation value of OTUs would fluctuate around the mean value of their neighborhood rather than depend on time. This allowed an estimation of the significant interval for the variation. Therefore, the stationarity analysis should be performed before analyzing time series data. It can be tested by detecting the presence or absence of unit root. The ADF-test was employed to verify if the time series data conformed to the stationary. If the original data was not subjected to stationarity, we used the difference, one minus another one to calculate the difference, to obtain the stationarity data. All relative abundance data of OTUs were filtered with the stationary test, while data that failed to go through the ADF test after two rounds of using difference would be summed in a separate file as nonstationary data. Although the relative abundances of OTUs may vary on a large scale, even seemingly without a mean value, the difference would be stationary in most situations. The operating reactors could be affected by many factors, which would shift the microbial community via stimulation of the metabolism of specific species.

### Granger causality

The Granger causality test is a statistical hypothesis test that determined the role of one time series in forecasting another one (Granger, 1969). Herein, the Granger causality is limited within interpreting the interaction of two OTUs that were subjected to the autoregressive–moving-average (ARMA) model. To *i*-th OTU, the ARMA model is shown as the equation:


$$z_{t\,i}=\delta +\sum_{l=1}^{q}\theta_{l}\,z_{\left(t-l\right)\,i}+v_{i}$$

*where v*_*i*_ is the random variation (white-noise series), δ is a constant, *z* is the abundance of species *i*, θ represents the parameters, and *q* is the model order. We simplified the equation for OTUs to the following format. Thus, we assumed the model for *i*-th OTU is X and the model for *j*-th OTU is Y. Both equations are as follows:


$$X_{t}=\delta +\sum_{l=1}^{q}\theta_{l}\,z_{\left(t-l\right)\,i}+v_{i}$$

$$Y_{t}=\delta +\sum_{l=1}^{q}b_{l}\,z_{\left(t-l\right)\,j}+\mu_{i}$$

where μ_*i*_ is the random variable. To know the interaction of X and Y, we assumed X and Y are interplays in their respective model predictions. The real data could be applied to the following equation:


$$X_{t}+b_{0}\,Y_{t}=\delta +\sum_{l=1}^{q}\theta_{l}\,X_{\left(t-l\right)\,i}+\sum_{l=1}^{q}b_{l}\,Y_{\left(t-l\right)\,j}+v_{i\,j}$$

$$Y_{t}+c_{0}\,X_{t}=\delta +\sum_{l=1}^{q}c_{l}\,X_{\left(t-l\right)\,i}+\sum_{l=1}^{q}d_{l}\,Y_{\left(t-l\right)\,j}+\mu_{j\,i}$$

where *b*_0_ and *c*_0_ are coefficients, and *b*_*l*_, *c*_*l*_, and *d*_*l*_ are the parameters. If *b*_0_ and *c*_0_ are not equal to 0 at the same time, this will be a model with instantaneous causality. In other words, *v*_*ij*_ and μ_*ji*_ would be the key to determining the Granger causality if the variation could be decreased when applying the *b*_0_ ≠ 0, and representing the *j*-th OTU can contribute to the prediction of *i*-th OTU, otherwise, there was no improvement in predicting *i*-th OTU with *j*-th OTU information. Therefore, the Granger causality can be tested by the ANOVA analysis to obtain a *p*-value. This relation between X and Y was termed as Granger causality, which implied X or Y can cause each other. Herein, the causal effects were attributed to the property of edges in the network.

### Network construction

All OTUs were filtered with two specific conditions such that OTUs with more than 80% non-zero values would be preserved. The residuals should comply with at least one relative abundance of individual OTU that reached more than 0.01% in all samples. The total number of OTUs was 98. The ADF test was applied to verify the stationarity of time series data and provide a proper lag for the next modeling process. The difference was calculated once OTUs failed in the ADF test, and the results of the difference were used to track the ADF test another time. All OTUs were reserved by the twice difference treatment. The time series matrix successfully inspected by the ADF test was used for the Granger test in pair. Before the operation of the Granger test, the order was determined by VAR (R package) (Pfaff, 2008). Subsequently, the lag was transferred to the Granger test. The *p*-value threshold of the Granger test was restricted by the following two methods. Due to the massively paired results, the links confirmed by the significance values could still cause statistical type I error, hence, we introduced Bonferroni multiple-comparisons procedure and false discovery rate (FDR) to correct the threshold. Bonferroni multiple-comparisons procedure was determined by the following equation:


α*=0.05(k2)

In the FDR test, all links that were selected by a 0.05 significant cut-off are reordered according to the magnitude of the *p*-value. FDR values were calculated by the following equation.


$$q_{i}=\text{k}\,\frac{p_{i}}{i}$$

$$F\,D\,R_{i}=\text{min}\,\left(q_{i},\cdots ,q_{k}\right)$$

where, *i* is the rank of the *p*-value in k links, which is the total links preserved by the previous threshold. The critical FDR value is normally 0.05. FDR has a great power to detect genuine positive effects, while the Bonferroni adjustment is more conservative and considers all comparisons to be statistically independent. The final file was transferred to Cytoscape software for further visualization and analysis. All analysis processes were completed with R, and several shiny apps had been built for this study (Stationary check^2^, Granger Causality network website^3^, Correlation network^4^, and MCCN^5^). The specific instruction for each app is provided in Supplementary Data S1. All raw R codes were deposited in the Github website.^6^

### Network indexes

The several properties of the causal network were referenced from the literature of Seth (2005), and termed as causal score (*cs*), causal density (*cd*), net causal flow (*ncf*), and causal reciprocity (*c*_*recip*_). Table 2 shows equations for all corresponding properties.

**TABLE 2:** Network indexes.

Name	Equation	Description
Causal score (*cs*)	$c\,s=\frac{{\text{n}}_{o}}{{\text{n}}_{o}+{\text{n}}_{i}}$	n_*o*_ is the outdegree, n_*i*_ is the indegree.
Causal density (*cd*)	$c\,d=\frac{n}{2\,N\,\left(N-1\right)}$	n is the total number of significant causal links preserved in the network file. N is the size of the network.
Graph efficiency (*ge*)	g⁢e=1-n-(N-1)(N2)	
Net causal flow (*ncf*)	*ncf* = n_*no*_ − n_*ni*_ n_*no*_ = *N*_*v*_ − n_*i*_ n_*ni*_ = *N*_*v*_ − n_*o*_	n_*no*_ is the net outdegree of a specific node. n_*i*_ is the net indegree of the individual node. *N*_*v*_ is the sum of all neighbors of specific OTU.
Causal reciprocity (*c*_*recip*_)	$c_{r\,e\,c\,i\,p}=\frac{{\text{n}}_{r\,e\,c\,i\,p}}{\text{n}}$	n_*recip*_ represents the number of reciprocal links in the total network. n is the value of total links.

As the network had been directed, outdegree and indegree represented the direction of edges within two nodes. The causal score (*cs*) was determined by the ratio of outdegree to total degrees of a specific node, reflecting the OTU influenced other OTUs rather than being influences. The causal score is defined as *cs* = the number of outdegrees divided by the number of indegrees in unweighted graphs (graphs in which all links are equivalent). If *cs* > 1, the corresponding OTU has active output, otherwise it is being passively influenced. The causal density is also termed as causal efficiency of the network, which, to some extent, represents the connectivity of the network. The net causal flow is the difference between the outdegree and indegree of each node, indicating the contribution of the individual node would be either active or passive. Herein, the active state represents the species intentionally affects others, while the passive indicates it is affected by others. Although causal flow is like a causal score, the former is intended to be independent of the quantity of balanced efferent and afferent connections. The causal reciprocity is the fraction of links with a directly reciprocal edge. Overall, the causal score and flow are applied to evaluate the role of each node, while the rest describes the whole network. Additionally, the supplemented indexes, including connectivity, centrality, stress centrality, etc., were analyzed with the Cytoscape software tool (Feng et al., 2017).

## Data availability statement

The original contributions presented in this study are included in the article/Supplementary material, further inquiries can be directed to the corresponding author.

## Author contributions

WC contributed to the conception and statistical analysis of the study. XH and HY contributed to manuscript revision, read, and approved the submitted version. TS revised the manuscript and interpreted the data for the work. All authors contributed to the article and approved the submitted version.

## Footnotes

1
https://itol.embl.de

2
https://caiweiwei.shinyapps.io/stationarycheck/

3
https://caiweiwei.shinyapps.io/causalnetwork/

4
https://caiweiwei.shinyapps.io/Cornetwork/

5
https://caiweiwei.shinyapps.io/combinenetwork/

6
https://github.com/weiwei12456/GCN

## Supplementary material

Supplementary Data S1 Description of MGCN apps family.

Supplementary Data S2 Stationary check results for 98 OTUs.

Supplementary Data S3 The *ncf* values of all OTUs in MGCN.

Supplementary Data S4 Heatmap of MCN and the network of BoMCCN.

Supplementary Data S5 The summary of species interaction around the hub OTU56.

Supplementary Data S6 MCCN of core-species.

Supplementary Data S7 Node index of the specific genera from MCCN.
